# Successful management of hyperammonemia with hemodialysis on day 2 during 5-fluorouracil treatment in a patient with gastric cancer: a case report with 5-fluorouracil metabolite analyses

**DOI:** 10.1007/s00280-020-04158-1

**Published:** 2020-10-03

**Authors:** Yoshinao Ozaki, Hirotaka Imamaki, Aki Ikeda, Mitsuaki Oura, Shunsaku Nakagawa, Taro Funakoshi, Shigeki Kataoka, Yoshitaka Nishikawa, Takahiro Horimatsu, Atsushi Yonezawa, Takeshi Matsubara, Motoko Yanagita, Manabu Muto, Norihiko Watanabe

**Affiliations:** 1 Department of Gastroenterology, Hirakata Kohsai Hospital, Osaka, Japan; 2 Department of Nephrology, Hirakata Kohsai Hospital, Osaka, Japan; 3 Faculty of Medicine, The University of Tokyo, Tokyo, Japan; 4 Department of Clinical Pharmacology and Therapeutics, Kyoto University Hospital, Kyoto, Japan; 5 Department of Therapeutic Oncology, Graduate School of Medicine, Kyoto University, Kyoto, Japan; 6 Department of Health Informatics, Kyoto University School of Public Health, Kyoto, Japan; 7 Department of Nephrology, Kyoto University Graduate School of Medicine, Kyoto, Japan

**Keywords:** 5-Fluorouracil, Hyperammonemia, Chronic renal failure, Pharmacokinetics

## Abstract

**Purpose:**

Hyperammonemia is an important adverse event associated with 5-fluorouracil (5FU) from 5FU metabolite accumulation. We present a case of an advanced gastric cancer patient with chronic renal failure, who was treated with 5FU/leucovorin (LV) infusion chemotherapy (2-h infusion of LV and 5FU bolus followed by 46-h 5FU continuous infusion on day 1; repeated every 2 weeks) and developed hyperammonemia, with the aim of exploring an appropriate hemodialysis (HD) schedule to resolve its symptoms.

**Methods:**

The blood concentrations of 5FU and its metabolites, α-fluoro-β-alanine (FBAL), and monofluoroacetate (FA) of a patient who had hyperammonemia from seven courses of palliative 5FU/LV therapy for gastric cancer were measured by liquid chromatography–mass spectrometry.

**Results:**

On the third day of the first cycle, the patient presented with symptomatic hyperammonemia relieved by emergency HD. Thereafter, the 5FU dose was reduced; however, in cycles 2–4, the patient developed symptomatic hyperammonemia and underwent HD on day 3 for hyperammonemia management. In cycles 5–7, the timing of scheduled HD administration was changed from day 3 to day 2, preventing symptomatic hyperammonemia. The maximum ammonia and 5FU metabolite levels were significantly lower in cycles 5–7 than in cycles 2–4 (NH3 75 ± 38 vs 303 ± 119 μg/dL, FBAL 13.7 ± 2.5 vs 19.7 ± 2.0 μg/mL, FA 204.0 ± 91.6 vs 395.9 ± 12.6 ng/mL, mean ± standard deviation, all *p* < 0.05). After seven cycles, partial response was confirmed.

**Conclusion:**

HD on day 2 instead of 3 may prevent hyperammonemia in 5FU/LV therapy.

**Electronic supplementary material:**

The online version of this article (10.1007/s00280-020-04158-1) contains supplementary material, which is available to authorized users.

## Introduction

5-Fluorouracil (5FU) is among the most commonly used chemotherapeutic agents in solid malignancy treatment, including gastrointestinal malignancies [1]; however, it causes various adverse events, such as myelosuppression, gastrointestinal toxicities, skin/subcutaneous tissue disorders, and hyperammonemia [2]. The management of these adverse events is vital for compliance with therapy.

Of particular interest in this study is hyperammonemia, which causes nausea, vomiting, tremors, and disturbances of consciousness. The incidence of hyperammonemia with any symptom is 5.7–8.7% among patients receiving a high-dose continuous infusion of 5FU (> 2000 mg/m^2^, > 24 h) [2–4]. Dehydration, renal dysfunction, constipation, infection, sarcopenia, and high-dose 5FU are some risk factors for hyperammonemia [5–8], and the condition is treated with 5FU discontinuation, hydration, lactulose (for constipation and inhibition of ammonia production and absorption in the intestinal tract), regimen change, and 5FU dose adjustment at the next cycle, among others [3–5].

Among patients with impaired renal function, 5FU can safely be administered without dose adjustments, as it is metabolized in the liver and not in the kidneys [9–12]. In a previous observational study, most cancer patients undergoing hemodialysis (HD) received 5FU without dose adjustments [13]. However, the elimination of α-fluoro-β-alanine (FBAL), a 5FU metabolite, is dependent on renal excretion [14]. 5FU is metabolized in the order of dihydrofluorouracil (FUDH), α-fluoro-β-ureidopropionic acid (FUPA), FBAL and monofluoroacetate (FA) [15] (Supplemental Fig. 1). FBAL accumulation has been observed among renal dysfunction patients with 5FU-associated hyperammonemia [15]. Hyperammonemia has previously been successfully managed with HD or hemodiafiltration [16, 17]; however, there is a lack of sufficient data on the modality and timing of renal replacement therapy during 5FU treatment.

**Fig. 1:**
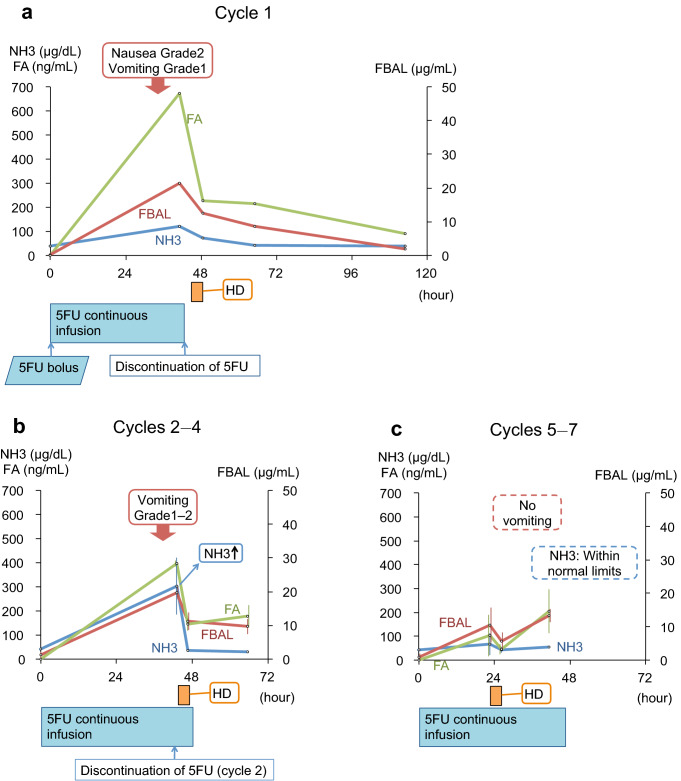
Clinical courses of cycle 1 **a**, cycles 2–4 average **b**, and cycles 5–7 average **c**. *HD* hemodialysis, *5FU* 5-fluorouracil, *FBAL* α-fluoro-β-alanine,*FA* monofluoroacetate

Here, we present a case of an advanced gastric cancer patient with chronic renal failure (CRF) who received 5FU/leucovorin (LV) infusion chemotherapy (simplified LV5FU2 regimen: 2-h leucovorin infusion at 200 mg/m^2^, followed by 400 mg/m^2^ bolus of 5FU, followed by 46-h continuous infusion of 2400 mg/m^2^ on day 1; repeated every 2 weeks) and developed hyperammonemia. To explore an appropriate HD schedule, we report the changes in the serum levels of 5FU and its metabolites in each cycle, and differences in the effects of hyperammonemia between HD administered on day 2 and day 3.

## Case presentation

An 81-year-old man was referred to Hirakata Kohsai Hospital with nausea and vomiting. He was diagnosed as having advanced gastric adenocarcinoma with pyloric stenosis and para-aortic lymph node metastases (cT3N2M1, cStage IV). He underwent gastrojejunostomy 9 days after admission. The patient also had advanced CRF with a 24-h creatinine clearance rate of 16.2 mL/min and received arteriovenous fistula surgery in preparation for chemotherapy, which would further aggravate renal function.

A simplified LV5FU2 regimen was initiated as a palliative chemotherapy a month after admission. In the first cycle (the dose of 5FU bolus/continuous infusion was reduced to 80% because of his old age, not because of CRF), the patient showed Grade 2 nausea and Grade 1 vomiting from the night of day 2 (nausea and vomiting were separately graded according to the Common Terminology Criteria for Adverse Events v.5.0). Continuous infusion was discontinued as the serum level of NH3 was high in the morning of day 3. Initially, HD was on standby in case it was needed. Based on previous reports [15–17], HD was selected and urgently performed on day 3 after 5FU discontinuation, and the patient quickly recovered from the symptoms. However, Grade 3 pneumonitis appeared on day 19.

From the second cycle, the interval between chemotherapy was prolonged to 3 weeks, considering the patient’s tolerability. Bolus 5FU was not administered, and the 5FU continuous infusion dose was decreased to 70% for the prevention of the hyperammonemia and pneumonitis that occurred in the first cycle. Nevertheless, in the second cycle, the patient experienced Grade 2 vomiting in the morning of day 3 accompanied by hyperammonemia. Continuous infusion was discontinued, and HD was performed on an urgent basis. The symptom quickly disappeared, and the level of NH3 returned to normal. Neither hyperammonemia nor suspicious symptoms appeared afterwards.

In cycles 3 and 4, the patient underwent scheduled HD on day 3 for hyperammonemia management, without 5FU dose changes. He experienced Grade 1 vomiting early in the morning of day 3 in cycle 3, and Grade 2 vomiting from the night of day 2 to the morning of day 3 in cycle 4. 5FU continuous infusion was completed after administration of HD.

In cycles 5–7, we aimed to prevent the onset of hyperammonemia by FBAL elimination, and, thus, set the HD administration timing to day 2, around 23 h after the start of 5FU continuous infusion which dose was maintained at 70%. The patient did not experience nausea or vomiting in cycles 5–7, and the serum NH3 levels were within the normal range except in cycle 5.

After the completion of seven cycles, partial response was attained; upper gastrointestinal endoscopy showed improvements in the degree of pyloric stenosis, and computed tomography (CT) found antral hypertrophy and para-aortic lymph node shrinkage. The serum level of carcinoembryonic antigen declined steadily throughout the courses (108 ng/mL before treatment, 32 ng/mL after cycle 4, 28 ng/mL before cycle 7). Renal function was preserved, and maintenance HD was deemed unnecessary during and after chemotherapy. Two weeks after cycle 7 completion, bilateral hearing loss appeared, and an examination revealed carcinomatous meningitis. Chemotherapy was discontinued, and the best supportive care was provided. The patient died 2 months later.

## Materials and methods

### Measurement

NH3 levels were quantified with FUJI DRI-CHEM SLIDE NH3-WII (FUJIFILM, Tokyo, Japan) (normal range 12–66 µg/dL). In each cycle, the serum was obtained and frozen before chemotherapy (day 0 or day 1), during continuous infusion, and before/after HD. The serum levels of 5FU and its metabolites, namely FUDH, FUPA, FBAL and FA, were measured using liquid chromatography tandem mass spectrometry (LCMS-8040, Shimadzu, Kyoto, Japan). The analytical methods were previously developed [15]. Calibration curves were constructed by an external calibration curve method. The lower limits of quantification were defined as the lowest concentration tested at which the coefficient of variation was less than 20%. Calibrator levels were 30, 100, 300, 1000, and 3000 ng/mL for 5FU, FUDH and FA; 30, 100, 300, 1000, 3000, 10,000, 30,000, and 100,000 ng/mL for FUPA and FBAL. The target 5FU concentration was considered as 434–543 ng/mL, as calculated from the optimal level of target area under the curve set from 20 to 25 mg·h/L during continuous 5FU infusion based on previous clinical study [18, 19].

### Statistical methods

The serum concentrations of NH3, 5FU, FUDH, FUPA, FBAL, and FA were compared using a two-sided Student’s *t* test with a significance level of 5%. All statistical analyses were performed using SPSS Statistics (version 21; IBM, Armonk, NY).

### Ethical consideration

This study was approved by the ethical review of Hirakata Kohsai Hospital (2019-013). Written informed consent for this case report was obtained both from the patient and his daughter.

## Results

Figure 1 and Supplemental Table 1 show the NH3, 5FU, FBAL, and FA concentration time courses (cycle 1: Fig. 1a, cycles 2–4: Fig. 1b, cycles 5–7: Fig. 1c). Supplemental Table 2 shows the comparisons of the concentrations of FUDH and FUPA.

### Cycle 1

Before HD on day 3, when nausea and hyperammonemia were present, the serum levels of 5FU, FBAL and FA were high. After continuous 5FU infusion was discontinued and symptomatic relief was achieved by HD, the concentrations decreased and never increased again.

### Cycles 2–4 (HD on day 3)

The doses of 5FU were the same in cycles 2–4 (dose: no bolus 5FU and 70% dose of 5FU continuous infusion). HD on day 3 led to the resolution of hyperammonemia (303 ± 119 µg/dL (mean ± standard deviation) to 36 ± 1 µg/dL), and the concentration of NH3 did not increase on day 4. The serum levels of FBAL and FA showed changes that were similar to those in NH3; they were high before HD on day 3 (19.7 ± 2.0 µg/mL and 395.9 ± 12.6 ng/mL), decreased just after HD (11.2 ± 2.6 µg/mL and 143.6 ± 26.3 ng/mL), and remained low on day 4.

The serum levels of 5FU before HD were higher (1784.7 ± 283.3 ng/mL) than the target 5FU concentration in cycles 2–4, although the 5FU dose was reduced. In cycles 3 and 4, 5FU continuous infusion was administered as planned before and after HD because scheduled HD was performed on day 3. Although the serum levels of 5FU after HD remained high, the symptoms disappeared during and after HD.

### Cycles 5–7 (HD on day 2)

The serum levels of FBAL and FA were high before HD on day 2 (10.5 ± 5.3 µg/mL and 102.6 ± 86.6 ng/mL), decreased just after HD (5.6 ± 2.8 µg/mL and 45.4 ± 22.0 ng/mL), and rose again in the morning of day 3 (13.3 ± 2.0 µg/mL and 204.0 ± 91.6 ng/mL). The serum levels of 5FU before HD were higher (1169.1 ± 387.0 ng/mL) than the target 5FU concentration, and were retained even after HD (1879.9 ± 904.3 ng/mL). HD did not lead to decreases in the serum levels of 5FU to levels below the target range.

### Comparison between cycles 2–4 (HD on day 3) and cycles 5–7 (HD on day 2)

Table 1 shows the comparison of the serum levels of NH3, FBAL, and FA between cycles 2–4 and cycles 5–7. The serum NH3, FBAL, and FA levels just before HD were 303 ± 119 µg/dL, 19.7 ± 2.0 µg/mL, and 395.9 ± 12.6 ng/mL, respectively, approximately 42 h after 5FU continuous infusion initiation in cycles 2–4, and 66 ± 46 µg/dL, 10.5 ± 5.3 µg/mL, and 102.6 ± 86.6 ng/mL, respectively approximately 23 h later in cycles 5–7; the levels showed a significant decrease (*p* = 0.032, 0.048, and 0.004). The maximum serum NH3, FBAL, and FA levels were 303 ± 119 μg/dL, 19.7 ± 2.0 μg/mL, and 395.9 ± 12.6 ng/mL, respectively, in cycles 2–4; these were observed just before HD on day 3. The corresponding maximum levels were 75 ± 38 μg/dL, 13.7 ± 2.5 μg/mL, and 204.0 ± 91.6 ng/mL, respectively, in cycles 5–7; again, significant decreases were noted (*p* = 0.034, 0.033, and 0.023).

**Table 1:** Comparisons of the concentrations of NH3, 5FU, and its metabolites (mean ± standard deviation)

	Cycles 2–4 before administration	Cycles 5–7 before administration		Cycles 2–4 day 3 before HD	Cycles 5–7 day 2 before HD		Cycles 2–4 max^a^	Cycles 5–7 max^b^		Cycles 2–4 day 3 after HD	Cycles 5–7 day 3	
NH3 (μg/dL)	41 ± 4	43 ± 3	(*p* = 0.497)	303 ± 119	66 ± 46	(*p* = 0.032)	303 ± 119	75 ± 38	(*p* = 0.034)	36 ± 1	53 ± 3	(*p* < 0.001)
5FU (ng/mL)	119.7 ± 38.3	93.8 ± 16.1	(*p* = 0.340)	1784.7 ± 283.3	1169.1 ± 387.0	(*p* = 0.090)	2240.4 ± 591.2	1879.9 ± 904.3	(*p* = 0.594)	1748.5 ± 1443.1^c^	1635.1 ± 741.6	(*p* = 0.909)
FBAL (μg/mL)	1.2 ± 0.9	0.9 ± 1.1	(*p* = 0.709)	19.7 ± 2.0	10.5 ± 5.3	(*p* = 0.048)	19.7 ± 2.0	13.7 ± 2.5	(*p* = 0.033)	11.2 ± 2.6	13.3 ± 2.0	(*p* = 0.312)
FA (ng/mL)	N.D.	N.D.		395.9 ± 12.6	102.6 ± 86.6	(*p* = 0.004)	395.9 ± 12.6	204.0 ± 91.6	(*p* = 0.023)	143.6 ± 26.3	204.0 ± 91.6	(*p* = 0.334)

The detection threshold of 5FU and its catabolites was 0.03 µg/mL (30 ng/mL)

*N.D.* not detected, *HD* hemodialysis, *5FU* 5-fluorouracil, *FBAL* α-fluoro-β-alanine, *FA* monofluoroacetate

^a^Day 3 after HD (5FU in cycles 3/4), and day 3 before HD (otherwise)

^b^Day 2 after HD (5FU), day 2 before HD (NH3/FBAL in cycle 5), and day 3 (otherwise)

^c^Note that in cycles 1/2, 5FU infusion was discontinued before HD due to Grade 2 nausea/vomiting

## Discussion

To the best of our knowledge, this is the first case report showing the effectiveness of HD on day 2 instead of day 3 for the prevention of 5FU-associated hyperammonemia. There have been few case reports of scheduled HD on day 2, while HD on day 3 has been common (Supplemental Table 3). These case reports did not include the comparison of symptoms by dialysis timing or metabolite measurements. In our patient, hyperammonemia was successfully treated with HD, alongside 5FU dose modifications. As the patient had advanced CRF, an arteriovenous fistula was created beforehand so that HD could be performed immediately if needed. Hyperammonemia presenting in cycles 1 and 2 was successfully managed by 5FU discontinuation and HD. With further careful management in later cycles, the patient could continue 5FU and showed only mild symptoms.

Additionally, changing the timing of HD from day 3 to day 2 prevented the occurrence of adverse events such as nausea, vomiting, and hyperammonemia. In previous reports, there is a lack of sufficient data on the modality and timing of renal replacement therapy during 5FU treatment, particularly in terms of avoiding hyperammonemia. Therefore, the present case, measuring 5FU metabolites at multiple points, may be beneficial in the management of 5FU-associated hyperammonemia in cancer patients with CRF.

HD was effective in the management of mild 5FU-associated hyperammonemia in our patient. In previous reports, symptomatic 5FU-related hyperammonemia was treated with 5FU discontinuation and therapeutic options such as hydration and lactulose use [3–5]. When these measures are insufficient, HD is efficacious, as the molecular weights (M) of FBAL and FA are sufficiently low (106 Da and 78 Da); HD is effective in the elimination of small molecules, especially those with size *M* ≤ 500 Da [20]. In this case, HD led to successful 5FU-related hyperammonemia management. Continuous hemodiafiltration was previously used in a patient with severe disease who was in a hyperammonemic coma [15, 17]. We were careful to keep our patient from dehydration and constipation throughout the cycles, so when hyperammonemia was present, we did not treat it except by HD.

HD administration on day 2 may be effective in the prevention of nausea, vomiting, and hyperammonemia as, in cycles 5–7 in our patient, there were no clinical symptoms of nausea/vomiting, unlike in cycles 2–4. The serum levels of NH3, FBAL, and FA before HD in cycles 5–7 (around 23 h after 5FU continuous infusion initiation) were significantly lower than those in cycles 2–4 (around 42 h after) (Table 1). It has been reported that serum FBAL and FA levels were elevated in 5FU-associated hyperammonemia [15]. In patients with renal dysfunction, 5FU treatment may cause hyperammonemia, as reduced FBAL renal excretion rates are associated with elevations in the levels of FBAL and its metabolite FA, which inhibits the tricarboxylic acid cycle [15] (Supplemental Fig. 1). In our patient, HD performed on day 3 was insufficient for the avoidance of FBAL accumulation and hyperammonemia, while that used on day 2 successfully reduced the maximum serum levels of FBAL and FA. This may have played a key role in the avoidance of nausea, vomiting, and hyperammonemia.

While it is a sensible concern that performing HD on day 2 during 5FU continuous infusion could lower serum 5FU levels, in cycles 5–7 in our case, the serum concentration of 5FU was retained above the target concentration and HD use did not decrease this concentration. Moreover, CT performed after cycle 7 revealed tumor shrinkage, indicating the clinical therapeutic effect of 5FU. Taken together, our results showed that HD administered on day 2 is a possible choice for the prevention of 5FU-associated hyperammonemia without reductions in the treatment effect in renal dysfunction patients.

Our study has some limitations. (1) While the patient’s renal function was heavily impaired, it was preserved to the extent that maintenance HD was deemed unnecessary. Dialysis was required for the elimination of FBAL and FA only during chemotherapy. Further research should focus on the management of chemotherapy among patients undergoing maintenance HD. (2) The data used in this report were obtained from one patient, although repeated measurements were performed. Further research is needed to confirm the generalizability of our results.

## Conclusion

5FU/LV was safely and effectively administered to a patient with gastric cancer and CRF. Hyperammonemia avoidance was achieved with the administration of HD on day 2 instead of day 3, alongside dose modifications.

## Electronic supplementary material

Below is the link to the electronic supplementary material.

Supplemental Fig. 1 The catabolic pathway of 5FU and the mechanism of hyperammonemia [15]. (1) Chronic renal failure impedes the renal excretion of FBAL. (2) The serum level of FBAL elevates. (3) Accumulated FBAL is metabolized to FA. (4) FA inhibits aconitase in TCA cycle. (5) TCA cycle fails to produce enough ATP and oxaloacetate for urea cycle. (6) Urea cycle fails to convert NH3 to urea. (7) The serum level of NH3 elevates. 5FU: 5-fluorouracil, DPD: dihydropyrimidine dehydrogenase, FUDH: dihydrofluorouracil, FUPA: α-fluoro-β-ureidopropionic acid, FBAL: α-fluoro-β-alanine, FA: monofluoroacetate, TCA: tricarboxylic acid, ATP: adenosine triphosphate (PDF 81 kb)

Supplementary file2 (PDF 184 kb)

Supplementary file3 (PDF 134 kb)

Supplementary file4 (PDF 176 kb)

## Data Availability

All data generated or analyzed during this study are included in this article and its supplementary information files.

## Code availability

All analyses were performed using SPSS Statistics (version 21; IBM, Armonk, NY).
