# Correction to: Successful management of hyperammonemia with hemodialysis on day 2 during 5‑fluorouracil treatment in a patient with gastric cancer: a case report with 5‑fluorouracil metabolite analyses

**DOI:** 10.1007/s00280-020-04193-y

**Published:** 2020-11-12

**Authors:** Yoshinao Ozaki, Hirotaka Imamaki, Aki Ikeda, Mitsuaki Oura, Shunsaku Nakagawa, Taro Funakoshi, Shigeki Kataoka, Yoshitaka Nishikawa, Takahiro Horimatsu, Atsushi Yonezawa, Takeshi Matsubara, Motoko Yanagita, Manabu Muto, Norihiko Watanabe

**Affiliations:** 1 Department of Gastroenterology, Hirakata Kohsai Hospital, Osaka, Japan; 2 Department of Nephrology, Hirakata Kohsai Hospital, Osaka, Japan; 3 Faculty of Medicine, The University of Tokyo, Tokyo, Japan; 4 Department of Clinical Pharmacology and Therapeutics, Kyoto University Hospital, Kyoto, Japan; 5 Department of Therapeutic Oncology, Graduate School of Medicine, Kyoto University, Kyoto, Japan; 6 Department of Health Informatics, Kyoto University School of Public Health, Kyoto, Japan; 7 Department of Nephrology, Kyoto University Graduate School of Medicine, Kyoto, Japan

## Correction to: Cancer Chemotherapy and Pharmacology (2020) 86:693–699 10.1007/s00280-020-04158-1

The article “Successful management of hyperammonemia with hemodialysis on day 2 during 5-fluorouracil treatment in a patient with gastric cancer: a case report with 5-fluorouracil metabolite analyses”, written by Yoshinao Ozaki · Hirotaka Imamaki · Aki Ikeda · Mitsuaki Oura · Shunsaku Nakagawa · Taro Funakoshi · Shigeki Kataoka · Yoshitaka Nishikawa · Takahiro Horimatsu · Atsushi Yonezawa · Takeshi Matsubara ·Motoko Yanagita · Manabu Muto and Norihiko Watanabe, was originally published Online First without Open Access. After publication in volume 86, issue 5, page 693–699 the author decided to opt for Open Choice and to make the article an Open Access publication. Therefore, the copyright of the article has been changed to © The Author(s) 2020 and the article is forthwith distributed under the terms of the Creative Commons Attribution 4.0 International License (https://creativecommons.org/licenses/by/4.0/), which permits use, sharing, adaptation, distribution and reproduction in any medium or format, as long as you give appropriate credit to the original author(s) and the source, provide a link to the Creative Commons licence, and indicate if changes were made.

The original article has been corrected.

