# Supplementary figures and images for: Successful management of hyperammonemia with hemodialysis on day 2 during 5-fluorouracil treatment in a patient with gastric cancer: a case report with 5-fluorouracil metabolite analyses
Source: Cancer Chemother Pharmacol. 2020 Oct 3;86(5):693–9. doi: 10.1007/s00280-020-04158-1 (PMC7595983; doi:10.1007/s00280-020-04158-1)

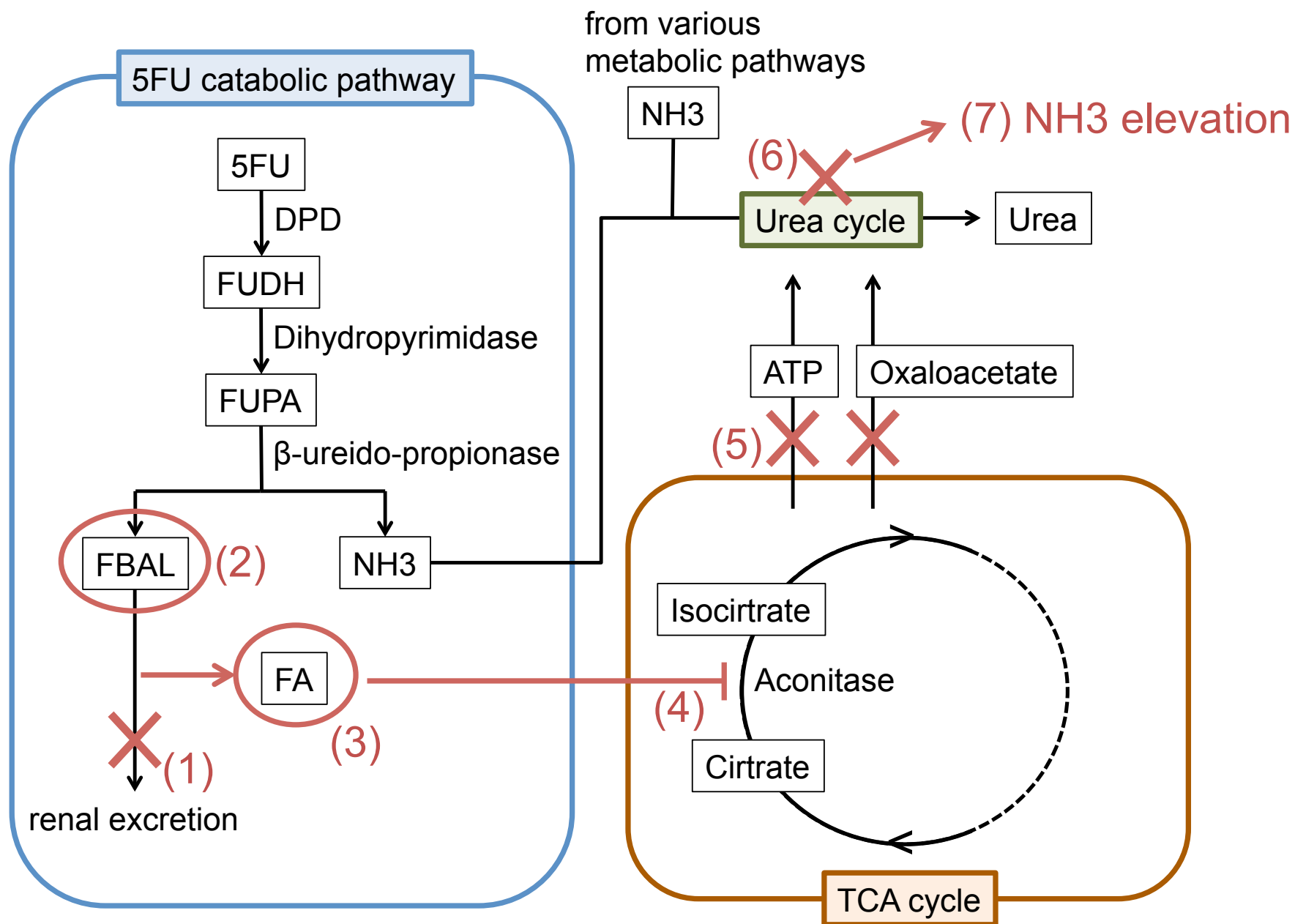

Supplement: Supplementary file 1 — Supplemental Fig. 1 The catabolic pathway of 5FU and the mechanism of hyperammonemia [15]. (1) Chronic renal failure impedes the renal excretion of FBAL. (2) The serum level of FBAL elevates. (3) Accumulated FBAL is metabolized to FA. (4) FA inhibits aconitase in TCA cycle. (5) TCA cycle fails to produce enough ATP and oxaloacetate for urea cycle. (6) Urea cycle fails to convert NH3 to urea. (7) The serum level of NH3 elevates. 5FU: 5-fluorouracil, DPD: dihydropyrimidine dehydrogenase, FUDH: dihydrofluorouracil, FUPA: α-fluoro-β-ureidopropionic acid, FBAL: α-fluoro-β-alanine, FA: monofluoroacetate, TCA: tricarboxylic acid, ATP: adenosine triphosphate (PDF 81 kb) [file 280_2020_4158_MOESM1_ESM.pdf]
