# Supplementary material for: Successful management of hyperammonemia with hemodialysis on day 2 during 5-fluorouracil treatment in a patient with gastric cancer: a case report with 5-fluorouracil metabolite analyses
Source: Cancer Chemother Pharmacol. 2020 Oct 3;86(5):693–9. doi: 10.1007/s00280-020-04158-1 (PMC7595983; doi:10.1007/s00280-020-04158-1)
Supplement: Supplementary file 2 — Supplementary file2 (PDF 184 kb) [file 280_2020_4158_MOESM2_ESM.pdf]

Supplemental Table 1 Time courses of the concentrations of NH3, 5FU, and its metabolites in each cycle

	Cycle 1						Cycle 2						Cycle 3						Cycle 4					
	NH3 (μg/dL)	5FU (ng/mL)	FUDH (ng/mL)	FUPA (μg/mL)	FBAL (μg/mL)	FA (ng/mL)	NH3 (μg/dL)	5FU (ng/mL)	FUDH (ng/mL)	FUPA (μg/mL)	FBAL (μg/mL)	FA (ng/mL)	NH3 (μg/dL)	5FU (ng/mL)	FUDH (ng/mL)	FUPA (μg/mL)	FBAL (μg/mL)	FA (ng/mL)	NH3 (μg/dL)	5FU (ng/mL)	FUDH (ng/mL)	FUPA (μg/mL)	FBAL (μg/mL)	FA (ng/mL)
before administration	40	385.8	52.9	N.D.	0.2	N.D.	36	162.5	N.D.	N.D.	0.2	N.D.	44	88.7	68.3	N.D.	1.7	N.D.	42	108.0	46.3	N.D.	1.8	N.D.
day 3 before HD	122	2845.9	1388.1	71.2	21.4	673.8	422	1557.7	1065.2	37.4	21.3	382.0	303	2102.2	699.6	54.4	17.4	406.7	185	1694.1	863.6	51.5	20.3	399.0
day 3 after HD	73	130.5†	44.1	21.0	12.5	226.1	35	82.1†	157.9	15.3	8.2	124.5	37	2582.1	530.1	26.7	12.4	173.6	35	2581.3	549.8	23.5	12.9	132.8
day 4	43	127.2	83.4	16.2	8.7	214.9	33	78.4	100.5	12.8	7.2	124.2	30	86.3	58.4	23.0	10.0	209.3	27	43.0	112.4	20.6	11.6	200.6
day 6	38	98.0	60.2	2.5	1.8	90.3																		

	Cycle 5						Cycle 6						Cycle 7					
	NH3 (μg/dL)	5FU (ng/mL)	FUDH (ng/mL)	FUPA (μg/mL)	FBAL (μg/mL)	FA (ng/mL)	NH3 (μg/dL)	5FU (ng/mL)	FUDH (ng/mL)	FUPA (μg/mL)	FBAL (μg/mL)	FA (ng/mL)	NH3 (μg/dL)	5FU (ng/mL)	FUDH (ng/mL)	FUPA (μg/mL)	FBAL (μg/mL)	FA (ng/mL)
before administration	41	80.7	252.1	N.D.	2.2	N.D.	41	88.9	N.D.	N.D.	0.2	N.D.	47	111.7	N.D.	N.D.	0.3	N.D.
day 2 before HD	119	1602.2	686.4	24.3	16.6	202.5	42	1047.7	346.5	26.8	7.7	50.2	38	857.3	112.4	24.3	7.3	55.1
day 2 after HD	56	2869.0	701.8	15.3	8.2	70.8	42	1675.2	100.9	12.5	6.0	34.6	31	1095.4	201.9	10.4	2.7	30.9
day 3	54	2472.2	1103.3	60.8	15.6	297.3	56	1372.6	805.7	43.4	12.0	200.5	50	1060.4	732.9	47.9	12.4	114.3

The detection threshold of 5FU and its catabolites was 0.03 μg/mL (30 ng/mL).
N.D. not detected, *HD* hemodialysis, *5FU* 5-fluorouracil, *FUDH* dihydrofluorouracil, *FUPA* α-fluoro-β-ureidopropionic acid, *FBAL* α-fluoro-β-alanine, *FA* monofluoroacetate
†Note that in cycle 1/2, 5FU infusion was discontinued before HD due to Grade 2 nausea/vomiting.

Article title: Successful management of hyperammonemia with hemodialysis on day 2 during 5-fluorouracil treatment in a patient with gastric cancer: a case report with 5-fluorouracil metabolite analyses

Journal name: *Cancer Chemotherapy and Pharmacology*

Author names: Yoshinao Ozaki, Hirotaka Imamaki, Aki Ikeda, Mitsuaki Oura, Shunsaku Nakagawa, Taro Funakoshi, Shigeki Kataoka, Yoshitaka Nishikawa, Takahiro Horimatsu, Atsushi Yonezawa, Takeshi Matsubara, Motoko Yanagita, Manabu Muto, Norihiko Watanabe
Affiliation and e-mail address of the corresponding author: Department of Gastroenterology, Hirakata Kohsai Hospital, Osaka, Japan; yoshinao@kuhp.kyoto-u.ac.jp
