# Supplementary material for: Successful management of hyperammonemia with hemodialysis on day 2 during 5-fluorouracil treatment in a patient with gastric cancer: a case report with 5-fluorouracil metabolite analyses
Source: Cancer Chemother Pharmacol. 2020 Oct 3;86(5):693–9. doi: 10.1007/s00280-020-04158-1 (PMC7595983; doi:10.1007/s00280-020-04158-1)
Supplement: Supplementary file 3 — Supplementary file3 (PDF 134 kb) [file 280_2020_4158_MOESM3_ESM.pdf]

Supplemental Table 2 Comparisons of the concentrations of FUDH and FUPA (mean ± standard deviation)

	Cycles 2–4 before administration	Cycles 5–7 before administration	Cycles 2–4 day 3 before HD	Cycles 5–7 day 2 before HD		Cycles 2–4 max (day 3 before HD)	Cycles 5–7 max (day 3)		Cycles 2–4 day 3 after HD	Cycles 5–7 day 3	
FUDH (ng/mL)	<48.2	<104.0	876.1 ± 183.1	381.8 ± 288.6	(*p*=0.066)	876.1 ± 183.1	880.6 ± 196.2	(*p*=0.978)	412.6 ± 220.8	880.6 ± 196.2	(*p*=0.052)
FUPA (μg/mL)	N.D.	N.D.	47.8 ± 9.1	25.1 ± 1.4	(*p*=0.013)	47.8 ± 9.1	50.7 ± 9.0	(*p*=0.712)	21.8 ± 5.9	50.7 ± 9.0	(*p*=0.010)

The detection threshold of FUDH and FUPA was 0.03 μg/mL (30 ng/mL).
N.D. not detected, *HD* hemodialysis, *FUDH* dihydrofluorouracil, *FUPA* α-fluoro-β-ureidopropionic acid

Article title: Successful management of hyperammonemia with hemodialysis on day 2 during 5-fluorouracil treatment in a patient with gastric cancer: a case report with 5-fluorouracil metabolite analyses
Journal name: *Cancer Chemotherapy and Pharmacology*
Author names: Yoshinao Ozaki, Hirotaka Imamaki, Aki Ikeda, Mitsuaki Oura, Shunsaku Nakagawa, Taro Funakoshi, Shigeki Kataoka, Yoshitaka Nishikawa, Takahiro Horimatsu, Atsushi Yonezawa, Takeshi Matsubara, Motoko Yanagita, Manabu Muto, Norihiko Watanabe
Affiliation and e-mail address of the corresponding author: Department of Gastroenterology, Hirakata Kohsai Hospital, Osaka, Japan; yoshinao@kuhp.kyoto-u.ac.jp
