# Supplementary material for: Successful management of hyperammonemia with hemodialysis on day 2 during 5-fluorouracil treatment in a patient with gastric cancer: a case report with 5-fluorouracil metabolite analyses
Source: Cancer Chemother Pharmacol. 2020 Oct 3;86(5):693–9. doi: 10.1007/s00280-020-04158-1 (PMC7595983; doi:10.1007/s00280-020-04158-1)
Supplement: Supplementary file 4 — Supplementary file4 (PDF 176 kb) [file 280_2020_4158_MOESM4_ESM.pdf]

Supplemental Table 3 Recent reported cases of scheduled HD during chemotherapy including 5FU continuous infusion for patients with chronic renal failure

Author	Year	Age	Sex	Primary lesion	Chemotherapy	5FU bolus dose[a]	5FU continuous dose[a]	Onset[b] cycle/day	NH3 (μg/dL)[c]	Cr (mg/dL)	scheduled HD timing[b]
Ohnishi et al.	2007	68	Female	sigmoid colon	FOLFOX4	300 mg/m^2 twice per cycle	500 mg/m^2 twice per cycle	cycle 1<br>day 2	Grade 2 vomiting<br>Grade 3 anorexia	7.1	day 1 (1 h after the start of 5FU continuous infusion) + day 3
Matoba et al.	2008	50s	Female	descending colon	mFOLFOX6	400 mg/m^2	2400 mg/m^2	–	–	–	day 1 (just after the infusion of oxaliplatin), HD were performed 3 times a week
Arai et al.	2008	65	Male	esophagus	5FU+CDDP	–	1250 mg/m^2	–	–	3.5	day 1 (30 min after the infusion of CDDP)
Katsumata et al.	2008	65	Male	cecum	mFOLFOX6	400 mg/m^2	2000 mg/m^2	cycle 1<br>day 8	Grade 3 fatigue<br>Grade 3 anorexia	6.49	day 1 (30 min after the infusion of oxaliplatin) + day 3
Fujita et al.	2008	77	Female	rectum	mFOLFOX6	400 mg/m^2	2400 mg/m^2	–	–	–	day 1 (just after the start of 5FU continuous infusion) + day 3 (48 h after)
Watayo et al.	2010	58	Male	rectum	mFOLFOX6	320 mg/m^2	1920 mg/m^2	–	–	8.59	day 1 (just after the infusion of oxaliplatin) + day 3 (48 h after)
Verwimp et al.	2010	73	Male	gastroesophageal junction	IRI+LV+5FU	–	2000 mg/m^2	–	–	–	day1 (before chemotherapy) + day3
Kuwabara et al.	2011	58	Female	sigmoid colon	mFOLFOX6<br>FOLFIRI/Bev	280 mg/m^2	1680 mg/m^2	–	–	–	day 1 (0–1 h after the infusion of oxaliplatin) + day 3 (48 h after)
Horimatsu et al.	2011	50	Male	sigmoid colon	mFOLFOX6+Bev	400 mg/m^2	2400 mg/m^2	–	–	–	day 1 (just after the infusion of oxaliplatin) + day 3 (48 h after)
Hoshino et al.	2012	47	Female	rectum	FOLFIRI+Bev	400 mg/m^2	2400 mg/m^2	–	–	–	before chemotherapy
Sato et al.	2013	67	Male	sigmoid colon<br>rectum	mFOLFOX6+Bev<br>sLV5FU2+Bev	400 mg/m^2	2400 mg/m^2	–	Grade 1 anorexia<br>Grade 1 nausea	–	mFOLFOX6+Bev: day 1<br>sLV5FU2+Bev: day 2
Matsuda et al.	2013	68	Male	sigmoid colon	FOLFOX4<br>FOLFIRI (+Bev)	400 mg/m^2 twice per cycle	600 mg/m^2 twice per cycle	–	–	10.2	day 1 + day 3
Bolonesi et al.	2014	55	Male	colon	FOLFIRI (+Bev)<br>mFOLFOX6	–	2400 mg/m^2	–	–	7.81	FOLFIRI: day 1 (before chemotherapy) + day3<br>mFOLFOX6: day 1 + day 3
Okamoto et al.	2014	64	Male	sigmoid colon	mFOLFOX6	320 mg/m^2	2800 mg/m^2	cycle 1<br>day 3	382	9.03	day 1 (1 h after the infusion of oxaliplatin) + day 3
Koike et al.	2015	71	Male	rectum	FOLFIRI<br>mFOLFOX6<br>LV-5-FU	280 mg/m^2	1680 mg/m^2	–	–	6.92	FOLFIRI: day 2<br>mFOLFOX6, LV-5-FU: not specified
Kobayashi et al.	2016	62	Male	cecum	mFOLFOX6+Pmab	400 mg.m^2	2400 mg/m^2	cycle 1<br>day 1	Grade 3 nausea<br>Grade 3 encephalopathy	8.73	day 1 (1 h after the infusion of oxaliplatin), HD were performed 3 times a week
Nishikawa et al.	2017	68	Male	cecum	mFOLFOX6+Bev	400 mg/m^2	2400 mg/m^2 (discontinued)	cycle 1<br>day 1	622	–	day 1 (between bolus 5FU and continuous 5FU)
van Berlo et al.	2018	77	Male	rectum	FOLFOX+Bev	340 mg/m^2	2040 mg/m^2	–	–	–	day 1 (just after the infusion of oxaliplatin) + day 3 (48 h after)
Yeung et al.	2018	59	Male	sigmoid colon	mFOLFOX	320 mg/m^2	2000 mg/m^2 (discontinued)	cycle 1<br>day 2	Grade 4 encephalopathy	12.6	day 1 (before chemotherapy)
Aimono et al.	2018	50s	Male	rectum	mFOLFOX+Bev	400 mg/m^2	2400 mg/m^2	cycle 1	Grade 1 anorexia	8.1	day 1 (just after the infusion of oxaliplatin), HD were performed 3 times a week
Wang et al.	2019	47	Female	descending colon	mFOLFOX	400 mg/m^2	2400 mg/m^2	–	–	–	day 1 (1 h after the infusion of oxaliplatin) + day 3 (59 h after)
Funasaka et al.	2019	65	Male	ascending colon	mFOLFOX6+Bev	400 mg/m^2	2400 mg/m^2	–	–	–	day 1 (just after the infusion of oxaliplatin) + day 3 (48 h after)
Funasaka et al.	2019	71	Male	rectum	mFOLFOX6+Bev	400 mg/m^2	2400 mg/m^2	–	–	–	day 1 (just after the infusion of oxaliplatin) + day 3 (48 h after)
Funasaka et al.	2019	71	Female	transverse colon	mFOLFOX6+Bev	400 mg/m^2	2400 mg/m^2	–	Grade 1 fatigue	–	day 1 (just after the infusion of oxaliplatin) + day 3 (48 h after)
Maruta et al.	2020	54	Male	cardia	FOLFOX	280 mg/m^2	1680 mg/m^2	–	–	3.65	day 1 (1 h after the infusion of oxaliplatin) HD were performed 3 times a week

[a] The maximum dose is written when the doses were different among cycles.
[b] The onset of hyperammonemia or the onset of possibly related adverse events to hyperammonemia (encephalopathy, seizure, headache, nausea, vomiting, anorexia, ataxia, fatigue, etc.)
[c] Possibly related adverse events to hyperammonemia are specified when the concentration of NH3 is not available.
5FU 5-fluorouracil, *CDDP* cisplatin, *LV* leucovorin, *IRI* irinotecan, *Bev* bevacizumab, *Pmab* panitumumab, *HD* hemodialysis, *CHDF* continuous hemodiafiltration, *CRRT* complete renal replacement therapy, *FOLFOX* folinic acid (leucovorin) + 5-fluorouracil + oxaliplatin, *mFOLFOX* modified FOLFOX, *FOLFIRI* folinic acid (leucovorin) + 5-fluorouracil + irinotecan

Article title: Successful management of hyperammonemia with hemodialysis on day 2 during 5-fluorouracil treatment in a patient with gastric cancer: a case report with 5-fluorouracil metabolite analyses
Journal name: *Cancer Chemotherapy and Pharmacology*
Author names: Yoshinao Ozaki, Hirotaka Imamaki, Aki Ikeda, Mitsuaki Oura, Shunsaku Nakagawa, Taro Funakoshi, Shigeki Kataoka, Yoshitaka Nishikawa, Takahiro Horimatsu, Atsushi Yonezawa, Takeshi Matsubara, Motoko Yanagita, Manabu Muto, Norihiko Watanabe
Affiliation and e-mail address of the corresponding author: Department of Gastroenterology, Hirakata Kohsai Hospital, Osaka, Japan; yoshinao@kuhp.kyoto-u.ac.jp
